# *T. cruzi* OligoC-TesT: A Simplified and Standardized Polymerase Chain Reaction Format for Diagnosis of Chagas Disease

**DOI:** 10.1371/journal.pntd.0000450

**Published:** 2009-06-02

**Authors:** Stijn Deborggraeve, Ximena Coronado, Aldo Solari, Ines Zulantay, Werner Apt, Pascal Mertens, Thierry Laurent, Thierry Leclipteux, Tim Stessens, Jean-Claude Dujardin, Piet Herdewijn, Philippe Büscher

**Affiliations:** 1 Department of Parasitology, Institute of Tropical Medicine Antwerp, Antwerp, Belgium; 2 Laboratory of Medicinal Chemistry, Rega Institute for Medicinal Research, Katholieke Universiteit Leuven, Leuven, Belgium; 3 Program of Molecular and Cellular Biology, Faculty of Medicine, University of Chile, Santiago, Chile; 4 Coris BioConcept, Gembloux, Belgium; National Institutes of Health, United States of America

## Abstract

**Background:**

PCR has evolved into one of the most promising tools for *T. cruzi* detection in the diagnosis and control of Chagas disease. However, general use of the technique is hampered by its complexity and the lack of standardization.

**Methodology:**

We here present the development and phase I evaluation of the *T. cruzi* OligoC-TesT, a simple and standardized dipstick format for detection of PCR amplified *T. cruzi* DNA. The specificity and sensitivity of the assay were evaluated on blood samples from 60 Chagas non-endemic and 48 endemic control persons and on biological samples from 33 patients, 7 reservoir animals, and 14 vectors collected in Chile.

**Principal Findings:**

The lower detection limits of the *T. cruzi* OligoC-TesT were 1 pg and 1 to 10 fg of DNA from *T. cruzi* lineage I and II, respectively. The test showed a specificity of 100% (95% confidence interval [CI]: 96.6%–100%) on the control samples and a sensitivity of 93.9% (95% CI: 80.4%–98.3%), 100% (95% CI: 64.6%–100%), and 100% (95% CI: 78.5%–100%) on the human, rodent, and vector samples, respectively.

**Conclusions:**

The *T. cruzi* OligoC-TesT showed high sensitivity and specificity on a diverse panel of biological samples. The new tool is an important step towards simplified and standardized molecular diagnosis of Chagas disease.

## Author Summary

Chagas disease (American trypanosomiasis) is caused by the protozoan parasite *Trypanosoma cruzi* and represents a major public health problem in Latin America. Furthermore, growing human population movements extend the disease distribution to regions outside the South American continent. Accurate diagnosis is crucial in patient care and in preventing transmission through blood transfusion, organ transplantation, or vertical transmission from mother to child. Routine diagnosis of *Trypanosoma cruzi* infection generally is based on detection of the host's antibodies against the parasite. However, antibody detection tests are liable to specificity problems and are of limited use in assessing treatment outcome and congenital infections. The introduction of the polymerase chain reaction (PCR) to amplify specific DNA sequences opened promising diagnostic perspectives. Despite its reported high sensitivity and specificity, broad use of the PCR technique in diagnosis of Chagas disease is hampered by its complexity and the lack of any standardization. We here present the development and evaluation of the *T. cruzi* OligoC-TesT, a simple and standardized dipstick format for detection of PCR amplified *T. cruzi* DNA. The new tool is an important step towards simplified and standardized molecular diagnosis of Chagas disease.

## Introduction

Accurate diagnosis of Chagas disease, the most important parasitic disease in the Americas [1], is challenging due to the latent character of the infection. Following a brief acute phase, infected individuals can progress to a lifelong chronic phase and about 30% will eventually develop the typical cardiac and intestinal complications that can be fatal [2]. *Trypanosoma cruzi*, the protozoan pathogenic agent, is naturally transmitted by blood sucking *Triatominae* bugs. Additional transmission routes include vertical transmission from mother to child, transfusion with infected blood and organ transplantation. Oral *T. cruzi* transmission by the consumption of contaminated food is linked to localized outbreaks of acute Chagas disease [3]. The *T. cruzi* species is genetically heterogeneous and has been classified in two major phylogenetic lineages, *T. cruzi* I and *T. cruzi* II, of which the latter is subdivided in 5 sublineages (IIa to IIe). *T. cruzi* I predominates in endemic countries north of the Amazon, whereas *T. cruzi* II is predominant throughout the Southern Cone countries of South America. *T. cruzi* I is associated with domestic and sylvatic transmission cycles, while IIa and IIc seem to be restricted to sylvatic cycles and IIb, IId and IIe to domestic cycles [4]. Both lineages are associated with cardiac lesions in human infection, but it seems that digestive tract lesions only occur in infection with *T. cruzi* II [5]. *T. cruzi* is closely related to *T. rangeli* that is apparently harmless to man but shares the same reservoir animals and vectors [6].

Acute infections, although rarely recognized because of the non-specific clinical manifestations, can be diagnosed by parasitological tests since the parasite load in the blood at that stage is generally high enough. Chronic infections, on the contrary, are associated with a very low parasite load in the blood and are therefore mainly diagnosed by means of antibody detection tests. However, antibody detection tests are liable to specificity problems due to cross-reaction with antibodies induced by other parasites, especially *Leishmania* and *T. rangeli* [7]. Hence, it is generally recommended that specimens are tested in two or three different immunodiagnostic tests, such as indirect immunofluorescence (IIF), indirect hemagglutination (IHA) and enzyme-linked immuno sorbent assays (ELISA). Furthermore, immunodiagnostic tests are less useful in testing the effectiveness of treatment and congenital infections. Xenodiagnosis is regularly used for parasite detection but results of this invasive test are only available after 1 to 3 months and the method shows generally low sensitivity with chronic infections [8]. Accurate tools to rapidly diagnose active infections, to assess treatment efficacy and to determine the impact of transmission control measures are needed. The introduction of the polymerase chain reaction (PCR) to amplify specific DNA sequences opened promising diagnostic perspectives. PCR has been used to detect *T. cruzi* in the blood of chronic chagasic patients [9], in congenital transmission [10], in patient follow-up after treatment [11] and in disease reactivation after heart transplantation [12]. Despite the reported high sensitivity and specificity, the PCR technique is restricted to high-level equipped laboratories. This is partly due to the laborious and time-consuming detection of the PCR products. Amplicons are generally detected by electrophoresis in agarose gel followed by U.V. illumination in the presence of the carcinogenic ethidium bromide. Quantitative real-time detection of PCR amplified *T. cruzi* DNA has been developed [13] and offers major advantages such as increased sensitivity, high-throughput analysis and single tube formats. However, the technique is complex and expensive. Besides the need for simplification of the DNA amplification assay, standardization is a crucial requirement for moving molecular tools towards patient diagnosis and disease control.

Oligochromatography (OligoC) provides a simple and rapid one-step dipstick format for detection of PCR products (Coris BioConcept, Gembloux, Belgium; Patent n° WO 2004/099438A1) [14]. After the PCR step, the detection step takes only 5 minutes and no other equipment than a water bath and pipette are needed. Controls for the PCR reaction and the chromatographic migration are incorporated in the assay. The technique has already been developed for diagnosis of human African trypanosomiasis [15], leishmaniasis [16], toxoplasmosis [17], schistosomiasis [18] and severe acute respiratory syndrome (SARS) [19], and high sensitivity and specificity were reported.

We here describe the development and phase I evaluation of a *T. cruzi* PCR-Oligochromatography test (*T. cruzi* OligoC-TesT), targeting a highly conserved satellite DNA sequence [20]. We assessed the diagnostic accuracy of the assay on a broad spectrum of biological samples from Chagas non-endemic and endemic control persons, infected children, adults, reservoir animals and vectors.

## Methods

### Experimental samples

*T. cruzi* Y (lineage IIb) epimastigotes were grown in glucose-lactalbumine-serum-hemoglobine (GLSH) medium [21] with 10% fetal calf serum at 26°C. At day 3 post-inoculation, parasites were counted in a Bürker counting chamber (Marienfeld, Germany). Tenfold dilution series of parasites ranging from 10,000 parasites/180 µl to 1 parasite/180 µl blood were made in human blood freshly taken on EDTA from a healthy volunteer. Non-spiked blood was used as negative control. Purified DNA from *T. cruzi* OPS21 Cl11, STC35R, IVV Cl3, X110/8, RN PCR 0 and VMV4, belonging to lineage I, IIa, IIb, IIc, IId and IIe, respectively, was obtained from the DNA reference bank at the Institute of Tropical Medicine Antwerp (ITMA), Belgium. DNA from 25 different *Trypanosoma rangeli* isolates (Table 1) and from *Leishmania donovani*, *Trypanosoma brucei gambiense*, *Mycobacterium tuberculosis*, *Schistosoma mansoni* and *Plasmodium falciparum* was obtained from other research groups. DNA concentrations were measured in the Nanodrop ND-1000 UV-Vis spectrophotometer (NanoDrop Technologies, Wilmington, USA) and the DNA was stored at −20°C.

**Table 1:** Code, origin, host, and amount of DNA detected by the *T. cruzi* OligoC-TesT of 25 *T. rangeli* isolates.

*T. rangeli* isolate	Origin	Host	DNA detected by OligoC-TesT
SJMC2	Bolivia	*Tamandua tetradactyla*	Not detected at 1 ng
PARAMA31	Venezuela	*Myrmecophaga tridactyla*	Not detected at 1 ng
RPLA4	Venezuela	*Rhodnius prolixus*	Not detected at 1 ng
A4	Venezuela	*Homo sapiens*	Not detected at 1 ng
B28P1A06P1C1	Venezuela	*Rhodnius prolixus*	Not detected at 1 ng
B28V1A06P2C3	Venezuela	*Rhodnius prolixus*	Not detected at 1 ng
CAN AC	Venezuela	*Canis lupus familiaris*	Not detected at 1 ng
C23	Colombia	*Rhodnius prolixus*	Not detected at 1 ng
C50	Colombia	*Rhodnius prolixus*	1 ng
Ev26	Colombia	*Aotus hivirgilus*	1 ng
R1271	Colombia	*Rhodnius prolixus*	1 ng
SJM30	Bolivia	*Tamandua tetradactyla*	1 ng
010	Panama	*Homo sapiens*	1 ng
P13	Venezuela	*Tamandua tetradactyla*	1 ng
RGB	Venezuela	*Canis lupus familiaris*	100 pg
B2	Venezuela	*Homo sapiens*	100 pg
SJMC1	Bolivia	*Tamandua tetradactyla*	100 pg
LEM2953	Guyana	*Bradypus tridactylus*	100 pg
LEM2946	Guyana	*Saguinus midas*	100 pg
LEM2947	Guyana	*Saguinus midas*	100 pg
LEM2952	Guyana	*Tamandua tetradactyla*	100 pg
LEM2963	Guyana	*Alouatta seniculus*	10 pg
LDG	Colombia	*Homo sapiens*	10 pg
CAS3	Colombia	*Homo sapiens*	10 pg
009	Panama	*Homo sapiens*	10 pg

### Biological samples

Ethical clearance for the study was obtained from the ethics committee of the Faculty of Medicine, University of Chile, Santiago, Chile. Written informed consent was given by the patients or guardians and from non-diseased persons. Research on biological samples from animals was conducted adhering to the institution's guidelines for animal husbandry.

#### Blood samples from Chagas non-endemic and endemic controls

DNA extracts from blood of 20 confirmed *T. brucei gambiense* infected persons from the Democratic Republic of the Congo (DRC), 20 confirmed *L. donovani* infected persons from Nepal and 20 confirmed *P. falciparum* infected persons from Zambia were obtained from the collection of the Department of Parasitology of the ITMA. Infections were confirmed by direct parasite detection in blood or tissue aspirates. Blood samples from 48 Chagas endemic control persons were obtained at the blood bank of the Clinical Hospital, University of Chile in 2004. Blood bank samples were classified as not infected with *T. cruzi* if negative in two different antibody detection tools (IIF and ELISA).

#### Blood samples from *T. cruzi* infected persons

2 ml whole blood from 6 children (0–10 years old) and 27 adults (>18 years old) infected with *T. cruzi* were collected in Chile (region IV), between the years 2000 and 2004. The blood was mixed with an equal volume of 6 M guanidine-HCl and 0.2 M EDTA, and 200 µl was subjected to DNA extraction. Persons were considered as infected with *T. cruzi* if positive in kDNA PCR amplifying the minicircle variable region followed by Southern blot hybridization with a *T. cruzi* specific probe, as described by Zulantay et al. [22]. For 20 of the 27 adult Chagas disease patients, clinical, serological and xenodiagnosis data were available. Six patients showed normal electrocardiogram (ECG) results, 11 showed prolonged QTC syndrome and one showed sinus tachycardia, while for 2 patients no ECG was done. All 20 patients were positive in two independent immunodiagnostic tests (IIF and ELISA). All 20 patients were negative in xenodiagnosis but 5 showed a positive result when the kDNA PCR was performed on the xenodiagnosis specimens.

#### Biological samples from reservoir animals and vectors

200 µl blood from 2 *Octodon degus* and 5 *Abrothrix olivaceus* rodents and the intestinal content from 14 *Mepraia spinolai* bugs were collected. All animals originated from Las Chinchillas National Reserve (Chile, region IV) and were classified as infected with *T. cruzi* based on positive results in kDNA PCR followed by Southern blot hybridization [22].

### DNA extraction

DNA from *T. cruzi* Y culture was extracted with the QIAamp DNA mini kit (Qiagen, Hilden, Germany). Spiked blood and blood from the endemic control persons were extracted using the QIAamp DNA blood mini kit (Qiagen, Hilden, Germany). DNA from blood of patients and animals was extracted with the EZNA blood DNA kit (Omega Bioteck, Inc., Doraville, GA). Intestinal content of the vectors was mixed with 200 µl PBS, boiled for 10 minutes and centrifuged at 10,000 g. The supernatant was used as DNA template.

### Primers and probes

**Figure 1:**
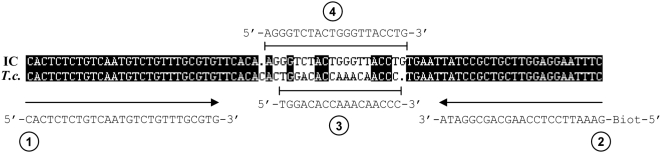
Presentation of the target DNA, primers and probes sequences. Alignment of the internal control (IC) DNA sequence and the *T. cruzi* OligoC-TesT DNA target sequence (*T.c.*) within the satellite DNA (consensus sequence of GenBank accession numbers AY519985 to AY520098). The forward primer Tc-Sat-F (1), the biotinylated (Biot) reverse primers Tc-Sat-R (2), the *T. cruzi* detection probe (3) and the IC detection probe (4) are shown.

An alignment of the internal control and target DNA, primers and probes sequences is presented in Figure 1. Primers, probes and internal control DNA were synthesized by Biomers.net (Ulm, Germany).

#### Primers

103 sequences of the 195 bp *T. cruzi* satellite DNA (GenBank accession numbers AY519985 to AY520098) were aligned using the “DNA man” software (Lynnon Corporation, Quebec, Canada). The sense primer Tc-Sat-F 5′-CACTCTCTGTCAATGTCTGTTTGCGTG-3′ and anti-sense primer Tc-Sat-R 5′-GAAATTCCTCCAAGCAGCGGATA-3′ were designed targeting a conserved 81 bp sequence within the *T. cruzi* satellite DNA. The anti-sense primer Tc-Sat-R was biotinylated at the 5′ end.

#### Internal control DNA

The internal control (IC) DNA sequence (81 bp) is identical to the *T. cruzi* target sequence except for a 20 bp central part. The IC central sequence was designed with the same length and the same GC content as the *T. cruzi* central sequence.

#### Probes

The *T. cruzi* detection probe (5′-TGGACACCAAACAACCC-3′) and the IC detection probe (5′-AGGGTCTACTGGGTTACCTG-3′) were designed to hybridize the *T. cruzi* and IC central sequences respectively. The detection probes were conjugated with gold particles using the procedure described in patent WO 2004/099438A1 [14]. The *T. cruzi* and IC migration control probes were synthesized with the reverse complement sequence of the respective detection probes.

### PCR amplification

The 50 µl PCR reaction mixture was prepared by adding 2.5 µl sample DNA and 1 unit of Hot Star Taq polymerase (Qiagen, Hilden, Germany) to 47.3 µl of *T. cruzi* Ampli-Mix (Coris BioConcept, Gembloux, Belgium). This pre-made PCR mix contains all components to allow PCR amplification, the primers Tc-Sat-F and Tc-Sat-R and the IC DNA at a concentration of 10^−18^ M. The commonly used dTTP was replaced by dUTP to allow elimination of carry-over contamination with uracyl-DNA N-glycosilase. An initial denaturation step at 94°C for 15 minutes to activate the Hot Star *Taq* polymerase was followed by 40 cycles of 94°C for 20 seconds, 65°C for 20 seconds and 72°C for 20 seconds, and a final extension at 72°C for 1 minute. Amplification was carried out in 200 µl thin-wall PCR tubes (Abgene, Epsom, United Kingdom) in a T3 thermocycler 48 (Biometra, Göttingen, Germany).

### Oligochromatography

#### Principle

**Figure 2:**
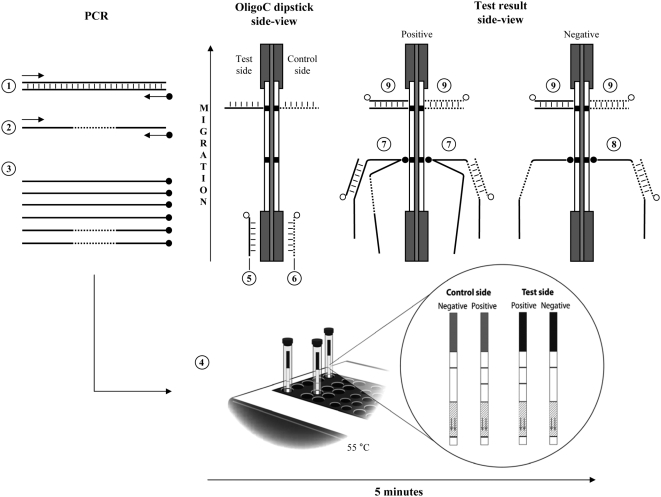
Schematic overview of the *T. cruzi* OligoC-TesT. Polymerase chain reaction (PCR) amplification of a sample containing *T. cruzi* DNA (1) is performed using a PCR mix containing single-stranded internal control (IC) template (2). The IC sequence is the same as the target sequence within the *T. cruzi* satellite DNA but with a specific internal sequence (dotted line). Both templates are amplified with the same primers of which the reverse primer is biotinylated (•). When the PCR and subsequent denaturation is completed, the PCR product solution contains single-stranded *T. cruzi* and IC DNA (3), and is mixed with an equal volume of migration buffer preheated at 55°C. The *T. cruzi* Oligo-Strip is dipped into the mixture and test results are read after 5 minutes migration at 55°C (4). During migration, the solution takes up the gold-labelled (○) detection probes. The *T. cruzi* detection probes (5) at the test side and the IC detection probes (6) at the control side hybridise with their respective amplicons. The biotinylated PCR products accumulate on the neutralite avidin lines at both sides of the dipstick and the *T. cruzi* and IC amplicons are visualized by their respective detection probes (7). In case of a negative sample, only the IC amplicon is present and visualized at the control side of the dipstick (8). The excess of detection probes migrate further and hybridise on the complementary probes coated at both sides of the dipstick as a control for migration (9).

The *T. cruzi* Oligo-Strip construction and principle is the same as the HAT-PCR-OC dipstick described by Deborggraeve et al. [15] but a migration control line was added to the test side. A schematic overview of the *T. cruzi* OligoC-TesT principle is presented in Figure 2. The IC line and the migration control lines determine whether the *T. cruzi* OligoC test is valid or invalid. An invisible migration control line indicates an invalid detection step, while an invalid PCR reaction is indicated by a negative IC line in combination with a negative test line. The latter may happen due to inhibitory factors in the extracted DNA. The assay is repeated when the control lines indicate failure of migration or of the PCR reaction. When a sample contains high concentrations of *T. cruzi* DNA, competition between the *T. cruzi* and the IC template DNA can result in an invisible IC control line but now combined with a strongly visible *T. cruzi* test line. In this case the test result is considered as a valid positive result.

#### Assay procedure

After PCR amplification the PCR product is denaturated at 94°C for 30 seconds and transferred immediately to ice. Forty microliters are mixed with an equal volume of OligoStrip running buffer preheated at 55°C, followed by dipping the *T. cruzi* Oligo-Strip into the solution. Test results are read after 5 minutes qualitatively. Whenever a test line appears that is visible by naked eye, the test is considered positive. Once test results are read the Oligo-Strips should not be stored for future reading as background may appear after drying of the strip.

## Results

### Analytical sensitivity

**Figure 3:**
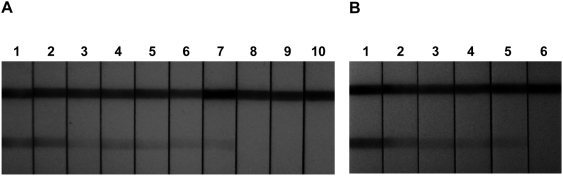
Analytical sensitivity of the *T. cruzi* OligoC-TesT. The upper and lower line are the migration control and the *T. cruzi* test line respectively. A. Test results on a serial dilution of *T. cruzi* strain Y DNA in water containing 0.1 mg/ml acetylated bovine serum albumine (BSA). Dipsticks 1–10: 1 ng, 100 pg, 10 pg, 1 pg, 100 fg, 10 fg, 1 fg, 0.1 fg, 0.01 fg and 0 fg per assay. B. Test results on a serial dilution of *T. cruzi* epimastigotes in naïve human blood. Dipsticks 1–6: 10,000; 1,000; 100; 10; 1 and 0 parasites in 180 µl blood.

The detection limit of the *T. cruzi* OligoC-TesT was evaluated on three independent tenfold serial dilutions of *T. cruzi* strain Y DNA in water containing 0.1 mg/ml acetylated bovine serum albumin (BSA). The lower detection limit was consistently 1 fg DNA per assay (Figure 3A) which is about 1/200 of the DNA content of one parasite. The analytical sensitivity was also evaluated on DNA extracted from three independent blood sample series spiked with decreasing numbers of living *T. cruzi* epimastigotes. The assay was able to detect 1 parasite in a 180 µl blood sample (Figure 3B), while non-spiked control blood samples always remained negative.

### Analytical specificity

The analytical specificity of the assay was evaluated on tenfold serial dilutions of DNA from *T. cruzi* strains representative for the 6 different lineages and on DNA from relevant non target pathogens. The *T. cruzi* OligoC-TesT was able to detect 1 fg DNA of the strains X110/8 (lineage IIc), RN PCR O (IId) and VMV4 (IIe), 10 fg DNA of the strains STC 35R (IIa) and IVV Cl3 (IIb), and 1 pg DNA of the strain OPS21 (I). Negative test results were observed with 5 ng purified DNA per test of *L. donovani*, *T. brucei gambiense*, *M. tuberculosis*, *S. mansoni* and *P. falciparum*. To assess the level of *T. rangeli* detection by the *T. cruzi* OligoC-TesT, tenfold serial dilutions of purified DNA from 25 different *T. rangeli* isolates were tested (Table 1). Four *T. rangeli* isolates could be detected at a DNA concentration of 10 pg per assay, 13 at a DNA concentration of 100 pg or 1 ng, while 8 isolates remained negative when tested at 1 ng DNA per assay.

### Specificity on Chagas non-endemic and endemic control blood samples

Negative results were observed when testing DNA extracts from blood of 20 *T. brucei gambiense* infected persons from DRC, 20 *L. donovani* infected persons from Nepal, 20 *P. falciparum* infected persons from Zambia, and 48 Chagas endemic control blood samples from Chile (Table 2). Hence, the specificity of the assay on 60 Chagas non-endemic and 48 endemic control blood samples was 100% with a 95% confidence interval [CI] scored by Wilson's method of 96.6% to 100% [23].

**Table 2:** Diagnostic accuracy of the *T. cruzi* OligoC-TesT on biological samples from Chagas non-endemic controls and endemic controls and from *T. cruzi* infected persons, reservoir animals and vectors.

Biological samples	#	Positive in *T. cruzi* OligoC	Specificity (95% CI)	Sensitivity (95% CI)
**Non-endemic controls**				
HAT patients (DRC)	20	0	100% (83.9%–100%)	
LEI patients (Nepal)	20	0	100% (83.9%–100%)	
MAL patients (Zambia)	20	0	100% (83.9%–100%)	
**Endemic controls (Chile)**				
Blood bank samples	48	0	100% (92.6%–100%)	
**Chagasic patients (Chile)**				
Children	6	4		66.7% (30%–90.3%)
Adults	27	27		100% (87.5%–100%)
**Infected animals (Chile)**				
*Octodon degus*	2	2		100% (34.2%–100%)
*Abrothrix olivaceus*	5	5		100% (56.6%–100%)
**Infected vectors (Chile)**				
*Mepraia spinolai*	14	14		100% (78.5%–100%)

Notes:

# number of samples.

95% CI 95% confidence interval (by Wilson's method).

DRC Democratic Republic of the Congo.

HAT human African trypanosomiasis.

LEI leishmaniasis.

MAL malaria.

### Sensitivity on *T. cruzi* infected patients, vectors and reservoir animals

The diagnostic accuracy of the *T. cruzi* OligoC-TesT was tested in 2007 on biological samples from 6 *T. cruzi* infected children and 27 infected adults, 2 infected *O. degus* rodents, 5 infected *A. alivaceus* rodents and 14 infected *M. spinolai* bugs (Table 2). All samples showed a positive test result except for 2 blood samples from children. Hence, the test showed a sensitivity of 66.7% (95% CI: 30%–90.3%), 100% (87.5%–100%), 100% (95% CI: 64.6%–100%) and 100% (95% CI: 78.5%–100%) on the specimens from infected children, adults, rodents and vectors, respectively. The two OligoC-TesT negative blood samples showed invalid results in two independent repetitions. Therefore, DNA was ethanol precipitated and washed as described elsewhere [15]. The purified DNA samples were retested in *T. cruzi* OligoC-TesT and valid negative test results were observed.

## Discussion

Antibody detection is routinely used for diagnosis of Chagas disease but suffers from specificity problems because of cross-reactivity with antibodies against other infectious agents such as *Leishmania* spp. and *T. rangeli*. Furthermore, immunodiagnosis is less useful in cure assessment after treatment and in diagnosis of congenital transmission. Xenodiagnosis is cumbersome, time consuming and invasive. Molecular detection of the parasite by the PCR technique is able to overcome most of these drawbacks since high sensitivity and specificity is combined with detection of DNA as surrogate for parasite detection. Despite the major advantages, PCR is still restricted to research applications and did not enter many diagnostic laboratories yet, which is mainly due to the complexity of the technique. Furthermore, the numerous in house diagnostic PCR assays and the neglect of any standardization hamper this promising technique to occupy a major position in clinical diagnosis. Next to patient management, PCR can play an important role as an accurate surrogate for parasite detection in epidemiological studies on reservoir animals and vectors.

In this study a molecular dipstick assay for simple, rapid and standardized detection of *T. cruzi* DNA was developed and evaluated. The *T. cruzi* OligoC-TesT consists of PCR amplification of a short sequence within the *T. cruzi* satellite DNA followed by a single step PCR product detection in dipstick format. The detection step is performed in 5 minutes and the only equipment needed is a pipette and water bath. The lower detection limits of the assay are 1 fg of purified *T. cruzi* strain Y DNA and 1 parasite in a 180 µl blood sample. This is similar to the detection limits of the primers Tcz1/Tcz2 and Diaz1/Diaz2 targeting the same 195 bp satellite DNA as reported by Moser et al. [24] and Diaz et al. [25], respectively. However, when we tested serial dilutions of DNA from *T. cruzi* strains representative for the 6 lineages we observed a 100 to 1000 times lower sensitivity on the *T. cruzi* strain of lineage I compared to the strains of lineage II. The lower sensitivity on *T. cruzi* lineage I is probably due to the lower copy number of the satellite repeats in lineage I as reported by Elias et al. [26]. This is confirmed by the fact that Diaz et al. also observed a lower sensitivity of their primers on DNA from *T. cruzi* I strains [25]. The test showed a positive signal when tested on purified DNA from *T. rangeli*. To assess the level of cross-reaction, serial dilution series of DNA of 25 different *T. rangeli* isolates were tested. Four of the 25 isolates could be detected at 10 pg DNA per test which is 10 times more than the required DNA of *T. cruzi* lineage I and 100 to 1000 times more than *T. cruzi* lineage II. Fourteen *T. rangeli* isolates required a higher amount of DNA to be detectable with the *T. cruzi* OligC-TesT and 8 remained negative at 1 ng DNA per assay. This is in contrast with Ochs et al. [27] and Virreira et al. [10] who reported no amplification of *T. rangeli* DNA by the Tcz1/Tcz2 primers. However, these authors used only one *T. rangeli* isolate and one DNA concentration. The observed cross-reaction with *T. rangeli* is not surprising since the satellite DNA has been described in *T. rangeli*. Breniere et al. [28] reported that the number of repeats of the 195 bp satellite DNA is far lower in *T. rangeli* than in *T. cruzi*, which might explain the higher detection threshold of the assay for *T. rangeli*. The variable *T. rangeli* detection levels are probably due to variable copy numbers of the satellite repeats within the *T. rangeli* population, as described for *T. cruzi* [26]. However, a parasitaemia of 10,000 *T. rangeli* parasites per ml of blood in human is unlikely since several studies reported no or very low *T. rangeli* multiplication in mammalian host cells [29],[30].

The specificity of the *T. cruzi* OligoC-TesT was estimated at 100% on blood samples from 60 Chagas non-endemic and 48 endemic control persons. Sensitivities of 93.9% on 33 infected patients and of 100% on 7 infected rodents and 14 infected vectors were observed. The high sensitivity on blood samples from adults is encouraging given the general low parasitaemia in the blood of such patients. However, we used a kDNA PCR followed by Southern blot as the main reference test instead of immunodiagnosis, that is currently the standard diagnostic technique for chronic Chagas disease. All 20 adult patients for which additional diagnostic test results were available showed positive immunodiagnostic test results but were negative in conventional xenodiagnosis, indicating the high sensitivity of PCR based parasite detection. Further evaluations are needed to estimate the diagnostic accuracy of the test on chronic Chagasic patients diagnosed on the basis of immunodiagnostic tools alone. The negative results on the two blood samples from children are unexpected since parasite loads are generally higher in children than in adults. The two samples showed initially invalid *T. cruzi* OligoC-TesT results, indicating inhibition of the PCR reaction. Therefore, the DNA was precipitated, washed and retested with the *T. cruzi* OligoC-TesT where after the tests became valid but negative. The DNA might have been lost during the multiple purification steps. The test showed high sensitivity on reservoir animals and vectors, which is promising regarding the coexistence of different *T. cruzi* lineages, including lineage I, at the collection site as reported by Rozas et al. [31].

In conclusion, the diagnostic accuracy on this diverse panel of biological samples indicates the potential of the *T. cruzi* OligoC-TesT. Further phase II and III evaluation studies are needed to assess the diagnostic sensitivity and specificity of the test on human target populations in different endemic regions. Persons with potentially cross-reacting diseases such as leishmaniasis should be included in the control group during future phase II evaluations. This new tool is a first step towards low-tech and user-friendly molecular diagnostics for *T. cruzi* detection. Besides the simplification, presentation of the *T. cruzi* OligoC-TesT as a self-containing kit with ready-to-use PCR mix and dipsticks offers excellent prospects for PCR standardization. Standardization of the PCR technique is crucial for its implementation as a reference parasite detection test for diagnosis and clinical studies. Chagas disease is routinely diagnosed by antibody detection but a simple and standardized PCR format can play an important role in situations were the use of immunodiagnosis is limited, such as congenital Chagas disease, patient follow-up after treatment and disease re-activation after organ transplantation. An additional potential niche for the *T. cruzi* OligoC-TesT may be patients with acute Chagas disease but negative test results in direct microscopy and patients with chronic Chagas disease but inconsistent or borderline immunodiagnostic test results. Isothermal amplification of the parasite's DNA through novel methods such as the loop mediated isothermal amplification (LAMP) [32] and the nucleic acid sequence based amplification (NASBA) [33] might further simplify the assay. Furthermore, standardized PCR formats to discriminate the 6 lineages of *T. cruzi* would be most welcome, since accurate lineage discrimination is important for disease and vector control programmes [34]. Additional standardization of the sampling procedures and DNA extraction is needed as well.

In conclusion, the *T. cruzi* OligoC-TesT represents a simplified and standardized PCR format for *T. cruzi* detection in biological samples. The innovative assay bears clear potential as a simplified molecular diagnostic tool for Chagas disease in midlevel-equipped laboratories.

## Supporting Information

Checklist S1 STARD Checklist
(0.10 MB DOC)
