# Supplementary figures and images for: T. cruzi OligoC-TesT: A Simplified and Standardized Polymerase Chain Reaction Format for Diagnosis of Chagas Disease
Source: PLoS Negl Trop Dis. 2009 Jun 2;3(6):e450. doi: 10.1371/journal.pntd.0000450 (PMC2685481; doi:10.1371/journal.pntd.0000450)

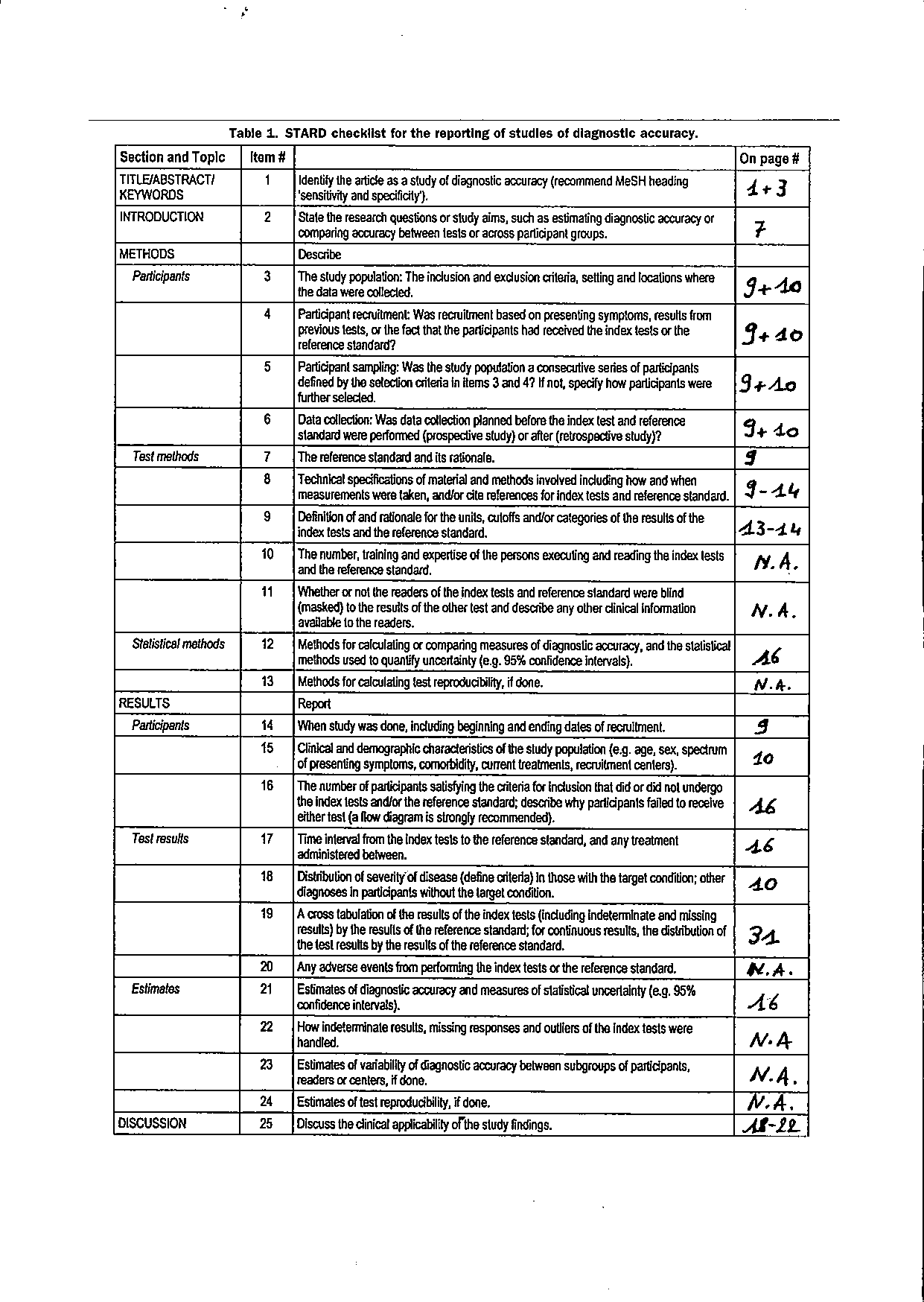


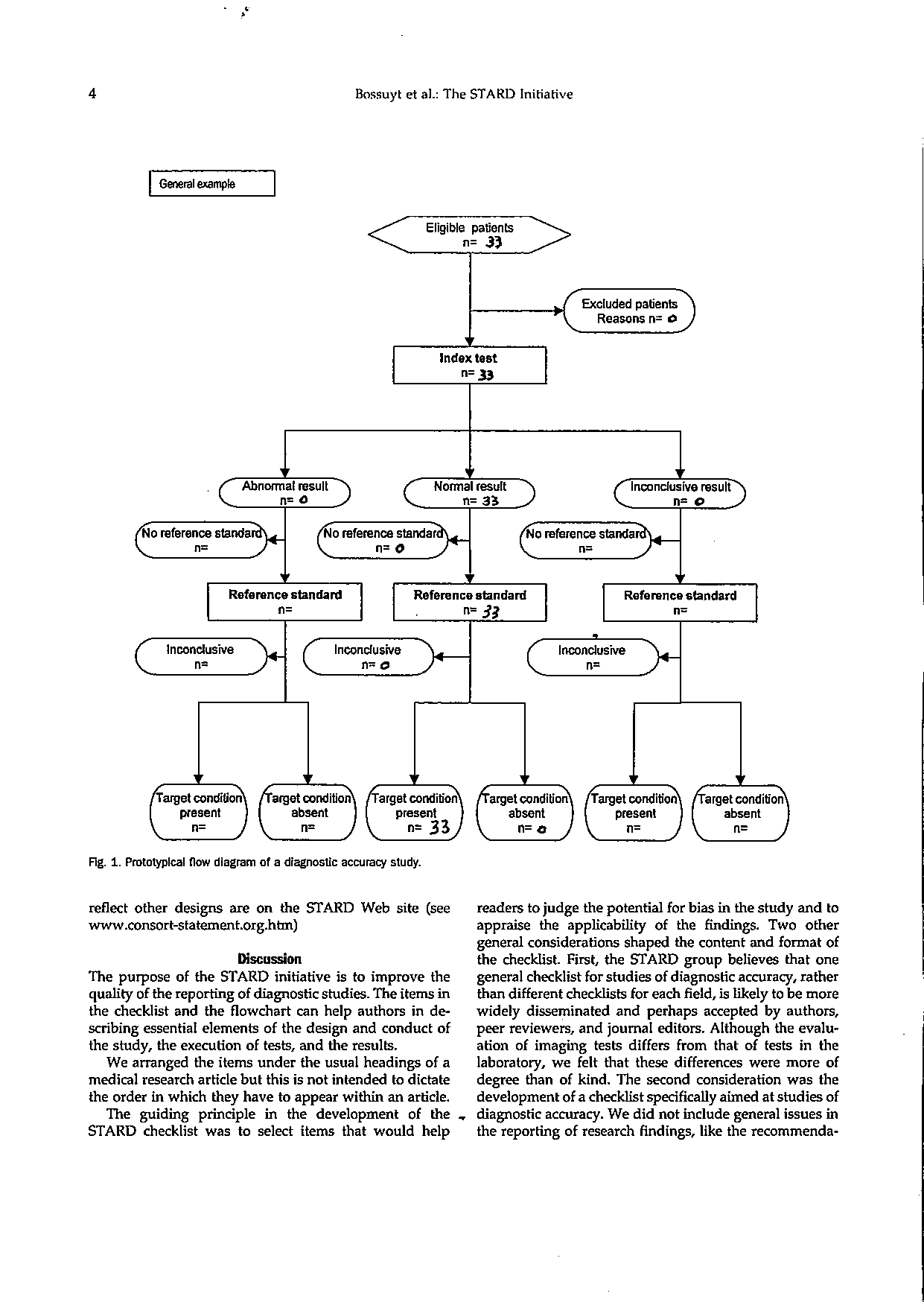

Supplement: Checklist S1 — STARD Checklist (0.10 MB DOC) [file pntd.0000450.s001.doc]
